# Mental Health Nurses' Perceived Appropriateness of Coercive Measures and Security Technologies in Psychiatric Settings: A National Cross‐Sectional Study

**DOI:** 10.1111/jnu.70097

**Published:** 2026-05-04

**Authors:** Giuliano Anastasi, Roberto Latina, Yari Longobucco, Alessandro Stievano, Stefano Bambi

**Affiliations:** ^1^ Department of Medicine and Surgery University of Enna Kore Enna Italy; ^2^ Department of Health Promotion, Mother and Childcare, Internal Medicine and Medical Specialties University of Palermo Palermo Italy; ^3^ Department of Health Sciences University of Florence Florence Italy; ^4^ Research and Development of Clinical Practice Unit Careggi University Hospital Florence Italy; ^5^ Department of Clinical and Experimental Medicine University of Messina Messina Italy

**Keywords:** attitudes, coercion, mental health, nurses, psychiatry, safety, technologies

## Abstract

**Introduction:**

Psychiatric settings are high‐risk environments for violence. Coercive measures (CMs) and security technologies (STs) can be used to ensure safety. However, limited evidence exists on how Italian mental health nurses (MHNs) perceive the appropriateness of such practices and the influencing factors. This study aimed to fill this gap.

**Design:**

Cross‐sectional study.

**Methods:**

An online survey collected sociodemographic data and validated measures of depression, anxiety, stress, stigma toward mental illness, and humanization of care. The perceived appropriateness of various CMs and STs was rated on a 5‐point Likert scale using a validated item set. Data were analyzed using descriptive statistics, bivariate tests, and multilevel mixed‐effects linear regression.

**Results:**

A total of 707 MHNs participated in the study. CMs were considered moderately appropriate (mean = 3.56 ± 0.92), with pharmacological restraint and locked‐door policies rated as more appropriate than physical restraint. STs were evaluated better (mean = 3.74 ± 0.95), with alarms and closed‐circuit television judged more appropriate than body‐worn cameras and metal detectors. CMs were considered less appropriate by non‐believers (*p* = 0.009), head nurses (*p* < 0.001), and those in non‐acute settings (*p* = 0.004), and more appropriate by those in Central Italy (*p* = 0.036), on daytime shifts (*p* = 0.042), and with higher stigma (*p* = 0.012). STs were considered less appropriate by males (*p* = 0.004), head nurses (*p* = 0.040), and more experienced MHNs (*p* < 0.001), and more appropriate by those in Southern Italy (*p* < 0.001) and in non‐acute settings (*p* < 0.001).

**Conclusion:**

MHNs consider CMs and STs moderately appropriate. Perceptions are influenced by both individual and contextual factors. Targeted training, anti‐stigma education, and inclusive policies are needed to ensure ethical and evidence‐based safety practices in psychiatric care.

**Clinical Relevance:**

Targeted education and training in mental health nursing, both continuing and post‐graduate, are essential to support cultural change among MHNs and ensure the appropriate use of CMs and STs. Integrating anti‐stigma initiatives and involving MHNs in policy development can strengthen clinical decision‐making and foster safer, more ethical, and person‐centred psychiatric care.

## 1 Introduction

Mental disorders are among the leading contributors to the global burden of disease, with a prevalence exceeding 970 million cases (GBD 2019 Mental Disorders Collaborators. 2022). Projections suggest a growth in this trend, with mental disorders potentially affecting nearly half of the global population in the future (McGrath et al. 2023).

Psychiatric units are essential for addressing this increasing demand, delivering care for individuals with severe psychological crises involving treatment refusal, high‐risk behaviors, or first‐time diagnoses (Bowers et al. 2009). However, these settings face significant safety challenges, including absconding, self‐harm, suicide, and violence (d'Ettorre and Pellicani 2017; Odes et al. 2021). For instance, up to 75% of patients are involved in aggressive incidents during hospitalization (Weltens et al. 2021), and 85% of healthcare workers experience aggression, 78% verbal assaults, and 37% sexual harassment (d'Ettorre and Pellicani 2017; Odes et al. 2021).

Therefore, ensuring the safety of patients, staff, and others has long been regarded as the foundation of psychiatric care (Bowers et al. 2010). Coercive measures (CMs) have historically been used for this purpose (Szasz 2007), involving interventions such as physical and pharmacological restraints, seclusion, and involuntary admission (Chieze et al. 2021; World Health Organization 2019). Nonetheless, the use of CMs in mental health services remains controversial due to ethical and clinical concerns (Chieze et al. 2021), including the potential for psychological harm (Cusack et al. 2018), physical injuries, or fatalities (Kersting et al. 2019). Despite international efforts to reduce their use (World Health Organization 2019), CMs remain prevalent, with worldwide prevalence rates of 25.7% for pharmacological restraint, 15.8% for seclusion, and 14.4% for physical restraints (Belayneh et al. 2024).

The persisting reliance on CMs underscores the need for alternative strategies to enhance safety (Doedens et al. 2020; Wong and Bressington 2022). National surveys have documented the use of various practices, including banning dangerous items, enforcing ward rules, and employing security technologies (STs) (Marhoon et al. 2019). In particular, STs have garnered increasing attention in recent literature (Anastasi and Bambi 2023), encompassing a wide range of measures, such as surveillance systems, including closed‐circuit television (CCTV) and body‐worn cameras (BWCs) (Foye et al. 2024; Revel et al. 2024), portable and fixed alarms (Gale et al. 2002), and metal detectors (Laidlaw et al. 2017). However, the implementation of STs in practice remains a debated topic. Practical barriers, such as the association of alarms with adverse events (Gale et al. 2002) and ethical concerns regarding video surveillance (Revel et al. 2024), emphasize the complexities involved in adopting these technologies. Moreover, evidence supporting their effectiveness is still limited (Anastasi and Bambi 2023; Revel et al. 2024), and questions persist about privacy violations, potential misuse, and the impact on the therapeutic relationship (Revel et al. 2024).

Mental health nurses (MHNs) serve as the primary staff in psychiatric settings (Delaney and Johnson 2014). Concerning their role, the literature highlights that maintaining safety and managing aggression are core responsibilities of MHNs (Delaney and Johnson 2014), as well as initiating and applying CMs (Happell and Harrow 2010). Consequently, a considerable body of research has explored MHNs' attitudes toward CMs (Doedens et al. 2020; Gugliotta et al. 2025; Happell and Harrow 2010; Laukkanen et al. 2020; Wong and Bressington 2022). In comparison, research on MHNs' views of STs remains at an early stage, reflecting the novelty of these interventions in psychiatric care (Anastasi and Bambi 2023), although emerging studies have begun to examine perspectives on specific devices, such as BWCs (Foye et al. 2024).

Similar to other mental health workers (Gugliotta et al. 2025), MHNs' perspectives on CMs are influenced by several individual and contextual factors, including demographic and professional characteristics such as age, gender, education level, and working experience, as well as emotional responses, personal beliefs, and values (Anastasi et al. 2024; Doedens et al. 2020; Happell and Harrow 2010; Laukkanen et al. 2020; Wong and Bressington 2022). In this regard, psychological factors appear particularly relevant. Emotional states have been shown to influence risk perception, emotional regulation, and clinical decision‐making related to the use of restrictive practices (Laukkanen et al. 2020; Wong and Bressington 2022). Anxiety and fear, in particular, have been identified as important drivers of decisions surrounding the implementation of CMs (Power et al. 2020). Moreover, greater psychological burden has been associated with more favorable attitudes toward CMs (Doedens et al. 2020), whereas positive psychological traits such as optimism and empathy—key components of the humanisation of care—may support less restrictive responses (Yang et al. 2014). Finally, stigma toward mental illness plays a critical role: professionals holding more negative attitudes toward mental illness may struggle more with emotional regulation and adopt more controlling approaches to care (Aluh et al. 2023), while higher levels of stigma have been shown to predict stronger endorsement of CMs (Steiger et al. 2023).

Given the complexities surrounding CMs and the increasing use of STs, further research is needed to understand MHNs' perspectives on these practices (Doedens et al. 2020; Happell and Harrow 2010; Laukkanen et al. 2020), especially in the Italian context, where empirical evidence remains limited (Gugliotta et al. 2025). In contemporary mental health care, ethical principles, legal standards, and humanized models of care increasingly emphasize least‐restrictive practice (Chieze et al. 2021; Hem et al. 2018; World Health Organization 2019), highlighting the relevance of exploring how MHNs evaluate the appropriateness of both CMs and STs. Moreover, the rapid integration of technological solutions introduces new ethical and professional challenges (Revel et al. 2024) that may shape staff perceptions. Lastly, considering psychological and attitudinal dimensions is essential to achieve a more comprehensive understanding of the factors that influence MHNs' evaluations of these practices. Therefore, investigating MHNs' perceived appropriateness of CMs and STs provides a valuable foundation for situating current safety policies and innovations within contemporary mental health nursing practice.

### 1.1 Aim

The primary aim of this study was to assess mental health nurses' perceptions of the appropriateness of various coercive measures and security technologies used in psychiatric settings. The secondary aim was to explore how these perceptions are influenced by sociodemographic, professional, and psychosocial factors, including depression, anxiety, stress, stigma toward mental illness, and the humanization of care.

## 2 Materials and Methods

### 2.1 Study Design

This study employed a descriptive cross‐sectional design to collect data from a nationwide sample of MHNs using an anonymous online survey. Ethical approval was obtained from the Local Ethics Committee and additional site‐specific ethics committees where the survey was distributed, following the institutional policies of the participating institutions. This study adheres to the Strengthening the Reporting of Observational Studies in Epidemiology (STROBE) guidelines (see Supporting Information). This study was part of a broader mixed‐methods research project (Anastasi et al. 2024).

### 2.2 Population and Sample Size

In Italy, nursing education is structured as a 3‐year university program culminating in a bachelor's degree in nursing. In these programs, mental health knowledge can be provided through interdisciplinary courses and field internships. However, there is a variability in nursing curricula nationwide, and although postgraduate courses in mental health nursing are available, they are not mandatory for employment in psychiatric settings. Based on this context, the study population comprised all nursing personnel employed in Italian mental health services, regardless of their level of education or specialized training in mental health nursing. In the Italian system, this comprises registered nurses and head nurses, who hold a university degree in nursing, as well as nursing aides, who are support personnel without a nursing degree and who assist with basic patient care activities under professional supervision. Participants were eligible if they met the following inclusion criteria: (1) employment as nursing personnel (i.e., registered nurses, head nurses, or nursing aides); (2) current employment in Italian mental health settings; (3) access to email and Internet for survey participation; (4) proficiency in Italian; and (5) provision of informed consent. Healthcare workers outside the nursing profession and nursing personnel working in non‐mental health settings were excluded.

According to the most recent data from the Italian Ministry of Health, public mental health services in Italy employ 15,522 MHNs, including 12,178 registered nurses and 3344 nursing aides; head nurses are classified within the registered nurse workforce (Italian Ministry of Health 2024). These data defined the size of the population from which the study sample was drawn. The required sample size was determined using Cochran's formula for prevalence studies: $n=\frac{t^{2}*\left[p*\left(1-p\right)\right]}{e^{2}}$ where: *n* is the required sample size, *t* represents the *t*‐value (1.96 for a 95% confidence level), *p* is the assumed prevalence, and *e* denotes the acceptable margin of error. In the absence of prior research, a prevalence of 50% was assumed to maximize the required sample size. Using a 95% confidence level and 5% margin of error, the minimum required sample size was 384 participants. To mitigate potential dropouts due to ineligibility or incomplete responses, a 30% increase was applied, resulting in a planned sample of 500 participants. To evaluate sample representativeness, the geographic distribution of participants was compared with national workforce statistics from the Italian Ministry of Health (2024). Percentage differences between the study sample and national estimates were calculated for each geographic area, and mean absolute differences were derived to provide an overall indicator of alignment between the sample and the national workforce distribution.

### 2.3 Data Collection Procedure

Participants were recruited using convenience and snowball sampling methods. Recruitment began with MHNs employed at the university hospital affiliated with the primary study centre. To ensure national representation, the research team compiled a contact list of MHNs employed in Italy by consulting the official websites of Mental Health Departments across the country. Recruitment emails encouraged potential participants, especially head nurses, to share the invitation within their professional networks, allowing for snowball sampling. The invitation email provided comprehensive information about the study, including its purpose, procedures, potential risks and benefits, privacy and confidentiality measures, the right to withdraw at any time, and a direct link to the online questionnaire. Data collection was conducted between 15 July and 31 October 2024 using an online self‐administered questionnaire hosted on Google Forms. The survey opened with a mandatory informed consent form that participants were required to complete. A brief initial screening ensured eligibility, and only those who met the inclusion criteria were allowed to proceed.

### 2.4 Measures

#### 2.4.1 Sociodemographic and Professional Variables

Sociodemographic variables included age, gender (female, male, or other), marital status (single, married, divorced, or widowed), ethnicity (Caucasian, Asian, or African), spiritual belief (Christian, Other, Agnostic, Atheist, or None), educational level (Professional Diploma, Bachelor's degree, First‐level master's degree, Master's degree in nursing and midwifery sciences, Second‐level master's degree, or Doctorate), academic education in mental health nursing (yes or no), and non‐academic education in mental health nursing (yes or no).

Professional variables included professional role (registered nurse, head nurse, or nursing aide), years of working experience, years of working experience in mental health, working area (North‐West, North‐East, Centre, South, or Islands), working setting (governance, acute, community, residential, or specialistic), contract type (permanent or fixed‐term), work schedule (full‐time or part‐time), and work pattern (rotating shift or daytime shift). Additional professional variables included perceived work safety, job satisfaction, intention to change the current work unit (i.e., transfer to another ward, service, or department within the same organization), and intention to leave the profession. Each variable was assessed using a single‐item measure rated on a 5‐point Likert scale ranging from 1 (“not at all”) to 5 (“very”). For each item, the mean score across participants was calculated, with higher scores indicating greater perceived safety, satisfaction, intention to change the current work units, or intention to leave the profession.

#### 2.4.2 Perceived Appropriateness of Coercive Measures and Security Technologies

Participants' perceptions of the appropriateness of CMs and STs were assessed using a set of items informed by interventions reported in previous studies (Marhoon et al. 2019). In the absence of a validated instrument, eight items were developed to evaluate the perceived appropriateness of three coercive measures (physical restraint, pharmacological restraint, and locked‐door policies) and five security technologies (closed‐circuit television, body‐worn cameras, fixed alarms, portable alarms, and metal detectors). Participants rated the appropriateness of each intervention on a 5‐point Likert scale ranging from 1 (“not at all appropriate”) to 5 (“very appropriate”). Example items included: “How appropriate do you consider the use of physical restraint?” and “How appropriate do you consider the use of closed‐circuit television?.” For each item, mean scores were calculated across all participants to summarize overall perceptions. Additionally, category‐level means were computed for coercive measures (three items) and security technologies (five items) by averaging the respective item scores at the sample level. The items were designed to capture general perceptions of appropriateness rather than context‐specific evaluations, allowing for an overall assessment of professionals' attitudes toward these interventions. Although the items did not constitute a psychometric scale, they underwent a structured development and validation process. Item generation was guided by the study's theoretical framework and an extensive literature review, followed by a focus group with a multidisciplinary panel comprising mental health head nurses, nursing academics, and a statistician to ensure conceptual relevance and clarity. Face and content validity were assessed using established methodologies. Satisfactory indices were obtained, with a Content Validity Ratio (CVR) ranging from 0.60 to 1.00, an Item‐Level Content Validity Index (I‐CVI) between 0.80 and 1.00, and a Scale‐Level Content Validity Index average (S‐CVI/Ave) of 0.90, all exceeding recommended thresholds. Face validity evaluation confirmed that the items were clear, relevant, and aligned with the study objectives. Full methodological details are available in the published protocol (Anastasi et al. 2024).

#### 2.4.3 Mental Health Outcomes (Depression, Anxiety, and Stress)

Mental health outcomes were assessed using the Italian version of the Depression, Anxiety, and Stress Scale (DASS‐21) (Bottesi et al. 2015). The scale comprises 21 items evenly distributed across three subscales: depression, anxiety, and stress. Example items include: “I felt that life was meaningless” and “I felt I was close to panic.” Participants rated the frequency of their symptoms over the previous week on a 4‐point Likert scale ranging from 0 (“never”) to 3 (“almost always”). Subscale scores were computed by summing the item scores within each dimension and multiplying the total by two, resulting in a range of 0–42 per subscale. Higher scores indicate more severe symptoms. Established cut‐off ranges were used for interpretation: depression (normal = 0–9, mild = 10–13, moderate = 14–20, severe = 21–27, extremely severe = ≥ 28), anxiety (normal = 0–7, mild = 8–9, moderate = 10–14, severe = 15–19, extremely severe = ≥ 20), and stress (normal = 0–14, mild = 15–18, moderate = 19–25, severe = 26–33, extremely severe = ≥ 34). The Italian version of the DASS‐21 has demonstrated excellent internal consistency, with Cronbach's alpha values of 0.82 for depression, 0.74 for anxiety, and 0.85 for stress (Bottesi et al. 2015).

#### 2.4.4 Stigma Toward Mental Illness

Stigma toward mental illness was assessed using the Italian version of the Opening Minds Stigma Scale for Healthcare Providers (OMS‐HC) (Destrebecq et al. 2018). This scale includes 12 items divided into two subscales: attitudes toward people with mental illness (five items) and attitudes toward disclosure of mental illness (seven items). Example items include: “I would be reluctant to seek help if I had a mental illness” and “There is little I can do to help people with mental illness.” Responses were scored on a 5‐point Likert scale ranging from 0 (“strongly disagree”) to 4 (“strongly agree”). Item 10 was reverse‐coded, and total scores were calculated by summing all item scores, generating a range of 0–48. Subscale scores ranged from 0 to 20 for attitudes toward people with mental illness and 0–28 for attitudes toward disclosure. Higher scores reflect greater stigma. The Italian version of the OMS‐HC showed strong internal consistency, with Cronbach's alpha values of 0.74 and 0.86 for the attitudes toward people with mental illness and attitudes toward disclosure of mental illness subscales, respectively (Destrebecq et al. 2018).

#### 2.4.5 Humanization of Care

The level of humanization in nursing practice was assessed using the Italian version of the Healthcare Professional Humanization Scale (HUMAS‐I) (Angelone et al. 2024). This scale consists of 19 items covering five dimensions: optimistic disposition, sociability, emotional understanding (three items each), self‐efficacy, and affection (five items each). Example items include: “I trust in the future with enthusiasm” and “I feel involved when I assist my patients.” Responses were recorded on a 5‐point Likert scale ranging from 1 (“never”) to 5 (“always”). Reverse‐coded items (items 15–19) were adjusted before calculating the total scores, which ranged from 19 to 95. Subscale scores ranged from 3 to 15 for optimistic disposition, sociability, and emotional understanding, and 5–25 for self‐efficacy and affection. Higher scores indicate greater humanization. The cut‐off values for interpretation were as follows: optimistic disposition (low = 0–11, mild = 12–14, high = 15), sociability (low = 0–13, mild = 14, high = 15), emotional understanding (low = 0–10, mild = 11, high = 12–15), self‐efficacy (low = 0–19, mild = 20–21, high = 22–25), affection (low = 21–25, mild = 16–20, high = 0–15), and humanization (low = 0–73, mild = 74–81, high = 82–95). The HUMAS‐I demonstrated good internal consistency, with Cronbach's alpha values of 0.82 for optimistic disposition, 0.70 for sociability, 0.81 for emotional understanding, 0.82 for self‐efficacy, and 0.87 for affection (Angelone et al. 2024).

### 2.5 Data Analysis

Data collected through Google Forms were downloaded and imported into Microsoft Excel for preliminary checks, including verification of data accuracy and identification of missing values. Questionnaires that were incomplete or had missing data were excluded from the analysis. Subsequently, the dataset was transferred to STATA software (version 16) for statistical analyses, which were conducted in consecutive steps. First, descriptive statistics, including frequencies, percentages, medians, means (*M*), standard deviations (SD), and quartiles, were used to summarize the characteristics of the study sample and scale scores. Differences between groups in terms of professional role were tested using ANOVA for continuous variables and chi‐square for discrete variables. Second, multilevel mixed‐effects linear regression models were employed to explore the relationships between perceived appropriateness (dependent variables) and potential predictors (independent variables). A forward stepwise approach was used for variable selection, wherein sociodemographic and professional variables with a significance level of *p* ≤ 0.10 in the univariate analyses were included in the multivariate model. Variables measuring levels of depression, anxiety, stress, stigma toward mental illness, and humanization of care were retained in the multivariate models, regardless of their significance, to ensure a comprehensive assessment of the influencing factors. A *p*‐value of < 0.05 was set as the threshold for statistical significance for all analyses.

## 3 Results

### 3.1 Characteristics of the Sample

A total of 761 MHNs participated in this survey. After excluding 54 incomplete questionnaires, the final sample comprised 707 respondents: 474 (67%) registered nurses, 116 (16.4%) head nurses, and 117 (16.5%) nursing aides. Detailed sample characteristics are presented in Table 1. The geographic distribution of the overall sample closely mirrored national mental health workforce estimates, with a mean absolute percentage difference of 2.8%, indicating no substantial geographic imbalances in the overall sample. A comparable geographic pattern was observed for registered nurses (mean absolute difference ≈3.0%). In contrast, larger regional discrepancies were identified among nursing aides (mean absolute difference ≈6.4%), primarily driven by their overrepresentation in southern regions (+16%) and underrepresentation in the North‐West (−9.6%). The full comparison is presented in Table 2.

**TABLE 1:** Sample characteristics.

Variable (range)	Total sample, *N*/mean (%/±SD)	Registered nurse, *N*/mean (%/±SD)	Head nurse, *N*/mean (%/±SD)	Nursing aide, *N*/mean (%/±SD)	*p*
**Participants**	707 (100)	474 (67)	116 (16.4)	117 (16.5)	
**Age, years**	50.16 (±9.70)	49.85 (±9.98)	52.66 (±7.94)	48.93 (±9.77)	**0.006**
**Gender**					0.618
Female	477 (67.5)	315 (66.5)	82 (70.7)	80 (68.4)	
Male	226 (32)	157 (33.1)	33 (28.4)	36 (30.8)	
Other	4 (0.5)	2 (0.4)	1 (0.9)	1 (0.9)	
**Marital status**					**0.003**
Single	174 (24.6)	129 (27.2)	20 (17.2)	25 (21.4)	
Married	396 (56)	260 (54.9)	79 (68.1)	57 (48.7)	
Divorced	116 (16.4)	74 (15.6)	12 (10.3)	30 (25.6)	
Widow	21 (3)	11 (2.3)	5 (4.3)	5 (4.3)	
**Ethnicity**					**0.035**
Caucasian	689 (97.5)	465 (98.1)	114 (98.3)	110 (94)	
Asian	14 (2)	8 (1.7)	1 (0.9)	5 (4.3)	
African	4 (0.6)	1 (0.2)	1 (0.9)	2 (1.7)	
**Spiritual belief**					0.144
Christian	549 (77.7)	362 (76.4)	87 (75)	100 (85.5)	
Other	14 (2)	9 (1.9)	4 (3.4)	1 (0.9)	
Agnostic	24 (3.4)	18 (3.8)	3 (2.6)	3 (2.6)	
Atheist	56 (7.9)	43 (9.1)	6 (5.2)	7 (6)	
None	64 (9.1)	42 (8.9)	16 (13.8)	6 (5.1)	
**Educational level**					**< 0.001**
Professional diploma	136 (19.2)	31 (6.5)	0 (0)	105 (89.7)	
Bachelor's degree	342 (48.4)	323 (68.1)	13 (11.2)	6 (5.1)	
First‐level master's degree	175 (24.8)	102 (21.5)	72 (62.1)	1 (0.9)	
Master's degree in nursing and midwifery sciences	48 (6.8)	18 (3.8)	26 (22.4)	4 (3.4)	
Second‐level master's degree	6 (0.8)	0 (0)	5 (4.3)	1 (0.9)	
**Academic education in mental health nursing**					**< 0.001**
No	531 (75.1)	356 (75.1)	71 (61.2)	104 (88.9)	
Yes	176 (24.9)	118 (24.9)	45 (38.8)	13 (11.1)	
**Non‐Academic education in mental health nursing**					**< 0.001**
No	71 (10)	41 (8.6)	2 (1.7)	28 (23.9)	
Yes	636 (90)	433 (91.4)	114 (98.3)	89 (76.1)	
**Working experience, years**	25.10 (±10.92)	24.92 (±10.80)	29.85 (±8.86)	21.14 (±11.56)	**< 0.001**
**Working experience in mental health, years**	14.81 (±11.41)	15.32 (±11.38)	17.96 (±11.83)	9.67 (±9.44)	**< 0.001**
**Working region**					**0.014**
North‐West	191 (27)	120 (25.3)	43 (37.1)	28 (23.9)	
North‐East	221 (31.3)	147 (31)	36 (31)	38 (32.5)	
Center	125 (17.7)	88 (18.6)	22 (19)	15 (12.8)	
South	126 (17.8)	83 (17.5)	10 (8.6)	33 (28.2)	
Islands	44 (6.2)	36 (7.6)	5 (4.3)	3 (2.6)	
**Working setting**					**< 0.001**
Governance	15 (2.1)	0 (0)	14 (12.1)	1 (0.9)	
Acute	278 (39.3)	194 (40.9)	37 (31.9)	47 (40.2)	
Community	245 (34.7)	184 (38.8)	39 (33.6)	22 (18.8)	
Residential	95 (13.4)	42 (8.9)	15 (12.9)	38 (32.5)	
Specialistic	74 (10.5)	54 (11.4)	11 (9.5)	9 (7.7)	
**Contract type**					**0.004**
Permanent	688 (97.3)	463 (97.7)	116 (100)	109 (93.2)	
Fixed‐term	19 (2.7)	11 (2.3)	0 (0)	8 (6.8)	
**Work schedule**					**0.035**
Full‐time	684 (96.7)	458 (96.6)	116 (100)	110 (94)	
Part‐time	23 (3.3)	16 (3.4)	0 (0)	7 (6)	
**Work shift**					**< 0.001**
Rotating shift	322 (45.5)	238 (50.2)	3 (2.6)	81 (69.2)	
Daytime shift	385 (54.5)	236 (49.8)	113 (97.4)	36 (30.8)	
**Perceived work safety (1–5)**	3.36 (±1.09)	3.21 (±1.12)	3.72 (±0.94)	3.61 (±0.98)	**< 0.001**
**Perceived job satisfaction (1–5)**	3.80 (±1.04)	3.71 (±1.05)	3.79 (±1.08)	4.21 (±0.83)	**< 0.001**
**Intention to change the current work unit (1–5)**	2.31 (±1.34)	2.36 (±1.33)	2.36 (±1.39)	2.03 (±1.31)	0.523
**Intention to leave the profession (1–5)**	2.08 (±1.38)	2.09 (±1.37)	2.47 (±1.52)	1.67 (±1.17)	**< 0.001**
**Appropriateness of coercive measures (1–5)**	3.56 (±0.92)	3.64 (±0.87)	3.07 (±1.01)	3.75 (±0.83)	**< 0.001**
Physical restraint (1–5)	3.08 (±1.25)	3.21 (±1.20)	2.47 (±1.15)	3.13 (±1.34)	**< 0.001**
Pharmacological restraint (1–5)	3.80 (±1.01)	3.88 (±0.97)	3.40 (±1.11)	3.91 (±1.03)	**< 0.001**
Locked‐door policy (1–5)	3.83 (±1.20)	3.85 (±1.20)	3.35 (±1.29)	4.23 (±0.95)	**< 0.001**
**Appropriateness of security technologies (1–5)**	3.74 (±0.95)	3.80 (±0.91)	3.36 (±1.05)	3.88 (±0.89)	**< 0.001**
Closed‐circuit television (1–5)	3.95 (±1.25)	3.99 (±1.25)	3.53 (±1.36)	4.24 (±1.03)	**< 0.001**
Body‐worn camera (1–5)	3.41 (±1.36)	3.48 (±1.33)	2.99 (±1.38)	3.55 (±1.44)	**0.001**
Fixed alarm (1–5)	4.03 (±1.07)	4.05 (±1.07)	3.76 (±1.12)	4.20 (±0.99)	**0.005**
Portable alarm (1–5)	4.01 (±1.13)	4.07 (±1.08)	3.72 (±1.26)	4.03 (±1.15)	**0.012**
Metal detector (1–5)	3.34 (±1.36)	3.46 (±1.30)	2.80 (±1.43)	3.38 (±1.40)	**< 0.001**
**Depression (0–42)**	7.19 (±8.09)	7.94 (±8.42)	7.17 (±8.31)	4.17 (±5.37)	**< 0.001**
**Anxiety (0–42)**	4.79 (±6.47)	5.41 (±6.81)	4.31 (±6.51)	2.76 (±4.20)	**< 0.001**
**Stress (0–42)**	12.28 (±8.82)	13.14 (±8.95)	12.58 (±8.40)	8.47 (±7.67)	**< 0.001**
**Stigma toward mental illness (0–48)**	12.30 (±6.54)	12.52 (±6.46)	12.18 (±7.08)	11.52 (±6.28)	0.323
Stigma toward people with mental illness (0–20)	6.58 (±3.78)	6.62 (±3.70)	6.94 (±4.08)	6.05 (±3.79)	0.179
Stigma toward disclosure of mental illness (0–28)	5.72 (±4.06)	5.90 (±4.05)	5.24 (±4.37)	5.47 (±3.79)	0.221
**Humanization of care (19–95**)	71.46 (±7.93)	70.92 (±7.99)	70.29 (±7.14)	74.82 (±7.62)	**< 0.001**
Optimistic disposition (3–15)	11.38 (±2.38)	11.24 (±2.39)	11.21 (±2.18)	12.08 (±2.44)	**0.002**
Sociability (3–15)	12.37 (±2.05)	12.31 (±2.05)	12.55 (±1.80)	12.47 (±2.27)	0.445
Emotional understanding (3–15)	11.57 (±2.18)	11.43 (±2.24)	11.48 (±1.95)	12.23 (±2.03)	**0.001**
Self‐efficacy (5–25)	19.46 (±3.01)	19.29 (±3.06)	19.15 (±2.83)	20.47 (±2.80)	**< 0.001**
Affection (5–25)	16.66 (±2.82)	16.63 (±2.89)	15.88 (±2.43)	17.54 (±2.67)	**< 0.001**

*Note:* Bold values indicate statistically significant values.

**TABLE 2:** Comparison of the geographic distribution of the study sample with that of the Italian national mental health workforce.

Geographic area	Nursing personnel				Registered nurses				Nursing aides			
	Sample, *N* (%)	Population, *N* (%)	Δ	Δ (mean)	Sample, *N* (%)	Population, *N* (%)	Δ	Δ (mean)	Sample, *N* (%)	Population, *N* (%)	Δ	Δ (mean)
North‐West	191 (27)	4618 (29.7)	−2.7	≈2.8	120 (25.3)	3498 (28.7)	−3.4	≈3.0	28 (23.9)	1120 (33.5)	−9.6	≈6.4
North‐East	221 (31.3)	3957 (25.5)	+5.8		147 (31)	2847 (23.4)	+7.6		38 (32.5)	1110 (33.2)	−0.7	
Center	125 (17.7)	3102 (20)	−2.3		88 (18.6)	2583 (21.2)	−2.6		15 (12.8)	519 (15.5)	−2.7	
South	126 (17.8)	2583 (16.6)	+1.2		83 (17.5)	2176 (17.9)	−0.4		33 (28.2)	407 (12.2)	+16	
Islands	44 (6.2)	1262 (8.1)	−1.9		36 (7.6)	1074 (8.8)	−1.2		3 (2.6)	188 (5.6)	−3	

*Note:* Δ represents the percentage difference between the study sample and national mental health workforce estimates. Δ (mean) denotes the mean absolute percentage difference across geographic areas and serves as an overall indicator of alignment between the study sample and the national workforce distribution. The national estimates were derived from the Italian Ministry of Health (2024). In national statistics, head nurses are classified within the registered nurse category; therefore, role‐specific comparisons were not possible.

#### 3.1.1 Sociodemographic and Professional Variables

The mean age of the participants was 50.16 years (±9.70), with head nurses being significantly older (52.66 ± 7.94, *p* = 0.006). The majority of the sample was female (67.5%, *n* = 477), with no significant sex differences across professional roles (*p* = 0.618). Regarding marital status, over half of the participants were married (56%, *n* = 396); however, significant differences emerged between groups (*p* = 0.003): registered nurses had the highest proportion of singles (27.2%, *n* = 129), head nurses had the highest proportion of married individuals (68.1%, *n* = 79), and nursing aides had the highest proportion of divorced individuals (25.6%, *n* = 30). The ethnic composition was predominantly Caucasian (97.5%, *n* = 689), although small but significant differences were observed among the groups (*p* = 0.035), with a slightly higher representation of non‐Caucasian participants among nursing aides. Most respondents identified as Christian (77.7%, *n* = 549), with no significant differences among the professional groups in terms of spiritual beliefs (*p* = 0.144). Concerning educational level, most participants had a bachelor's degree (48.4%, *n* = 342); however, education differed significantly between roles (*p* < 0.001). While most nursing aides had a professional diploma (89.7%, *n* = 105), most registered nurses held a bachelor's degree (68.1%, *n* = 323), and the majority of head nurses completed a first‐level master's degree (62.1%, *n* = 72). Formal academic education in mental health nursing was reported by 24.9% (*n* = 176) of the sample, with head nurses being significantly more trained (38.8%, *n* = 45; *p* < 0.001). In contrast, non‐academic education in mental health nursing was widespread (90%, *n* = 636), although significantly less common among nursing aides (76.1%, *n* = 89; *p* < 0.001).

Participants had, on average, 25.10 (±10.92) years of work experience, with head nurses having significantly more years of work experience (29.85 ± 8.86, *p* < 0.001). Similarly, the average work experience in mental health was 14.81 years (±11.41), with nursing aides reporting significantly lower tenure (9.67 ± 9.44, *p* < 0.001). Participants were primarily employed in the North‐East (31.3%, *n* = 221) and North‐West (27%, *n* = 191) regions of Italy. The group distribution across roles varied significantly by region (*p* = 0.014). Employment settings also differed significantly (*p* < 0.001): 39.3% (*n* = 278) of participants worked in acute care, 34.7% (*n* = 245) in community care, and 13.4% (*n* = 95) in residential care. Registered nurses were more present in community settings (38.8%, *n* = 184), head nurses were more likely to work in governance (12.1%, *n* = 14), and nursing aides were most represented in residential care (32.5%, *n* = 38). Almost all respondents held permanent contracts (97.3%, *n* = 688) and worked full‐time (96.7%, *n* = 684). However, nursing aides were more likely to be on fixed‐term contracts (6.8%, *n* = 8; *p* = 0.004) and part‐time schedules (6%, *n* = 7; *p* = 0.035). Most participants worked the daytime shift (54.5%, *n* = 385), with significant variations across roles (*p* < 0.001). Generally, head nurses worked on daytime shifts (97.4%, *n* = 113), whereas registered nurses (50.2%, *n* = 238) and nursing aides (69.2%, *n* = 81) worked on rotating shifts.

Overall, the participants reported moderate levels of perceived work safety (3.36 ± 1.09) and job satisfaction (3.80 ± 1.04). However, these outcomes varied significantly across different professional roles. Registered nurses perceived the lowest safety (3.21 ± 1.12, *p* < 0.001), whereas nursing aides expressed the highest job satisfaction (4.21 ± 0.83; *p* < 0.001). Intentions to change the current work unit were generally low across the sample (2.31 ± 1.34), with no significant differences among the groups (*p* = 0.523). In contrast, intention to leave the profession, despite being low in the sample (2.08 ± 1.38), was significantly lower among nursing aides (1.67 ± 1.17; *p* < 0.001).

#### 3.1.2 Perceived Appropriateness of Coercive Measures and Security Technologies

Participants generally rated both the CMs and STs as moderately appropriate. The overall mean score for the CMs was 3.56 (±0.92). Among specific measures, locked‐door policies (3.83 ± 1.20) and pharmacological restraint (3.80 ± 1.01) were perceived as more appropriate, whereas physical restraint had the lowest score (3.08 ± 1.25). The perceived appropriateness of STs was slightly higher, with a mean score of 3.74 (±0.95). Fixed alarms (4.03 ± 1.07) and portable alarms (4.01 ± 1.13) were considered more appropriate than CCTV (3.95 ± 1.25). In contrast, BWCs (3.41 ± 1.36) and metal detectors (3.34 ± 1.36) received lower average ratings.

Significant differences in perceived appropriateness were observed across professional roles. Head nurses consistently reported the lowest ratings for both CMs (3.07 ± 1.01, *p* < 0.001) and STs (3.36 ± 1.05, *p* < 0.001). In particular, they rated physical restraint (2.47 ± 1.15) and metal detectors (2.80 ± 1.43) as significantly less appropriate than other professional groups (*p* < 0.001). Similarly, they gave notably lower evaluations for pharmacological restraint (3.40 ± 1.11, *p* < 0.001), locked‐door policy (3.35 ± 1.29, *p* < 0.001), CCTV (3.53 ± 1.36, *p* < 0.001), and BWCs (2.99 ± 1.38, *p* = 0.001), although these were still considered moderately appropriate. In contrast, nursing aides reported the highest levels of perceived appropriateness for both CMs (3.75 ± 0.83, *p* < 0.001) and STs (3.88 ± 0.89, *p* < 0.001). This trend was particularly marked for locked‐door policies (4.23 ± 0.95, *p* < 0.001), pharmacological restraints (3.91 ± 1.03, *p* < 0.001), CCTV (4.24 ± 1.03, *p* < 0.001), and fixed alarms (4.20 ± 0.99, *p* = 0.005). Registered nurses generally expressed intermediate levels of perceived appropriateness but rated some interventions more favorably than other professionals. These included physical restraints (3.21 ± 1.20, *p* < 0.001), portable alarms (4.07 ± 1.08, *p* = 0.012), and metal detectors (3.46 ± 1.30, *p* < 0.001).

#### 3.1.3 Mental Health Outcomes, Stigma Toward Mental Illness, and Humanization of Care

The participants reported overall low levels of psychological distress. The average scores were 7.19 (±8.09) for depression, 4.79 (±6.47) for anxiety, and 12.28 (±8.82) for stress. However, statistically significant differences were observed across the professional roles (*p* < 0.001 for all three variables). Nursing aides consistently reported the lowest levels of psychological distress, with mean scores of 4.17 (±5.37) for depression, 2.76 (±4.20) for anxiety, and 8.47 (±7.67) for stress.

Stigma toward mental illness was moderate across the sample population. The overall stigma score averaged 12.30 (±6.54), with subscale means of 6.58 (±3.78) for stigma toward people with mental illness and 5.72 (±4.06) for stigma related to the disclosure of mental illness. No statistically significant differences emerged between professional roles regarding total stigma or its subdimensions.

The mean humanization score was 71.46 (±7.93), reflecting a generally low level of humanization. Significant differences were found between professional roles (*p* < 0.001), with nursing aides reporting the highest humanization (74.82 ± 7.62). Regarding dispositional traits related to humanization, emotional understanding (11.57 ± 2.18) and affection (16.66 ± 2.82) were mild, while optimistic disposition (11.38 ± 2.38), sociability (12.37 ± 2.05), and self‐efficacy (19.46 ± 3.01) were low, although they were nearing the mild thresholds. Except for sociability (*p* = 0.445), significant differences were observed across professional roles for all traits. Nursing aides consistently reported the highest levels of emotional understanding (12.23 ± 2.03, *p* = 0.001), optimistic disposition (12.08 ± 2.44, *p* = 0.002), self‐efficacy (20.47 ± 2.80, *p* < 0.001), and affection (17.54 ± 2.67, *p* < 0.001).

### 3.2 Determinants of Perceived Appropriateness of Coercive Measures and Security Technologies

The determinants of the perceived appropriateness of CMs and STs were examined using univariate and multivariate mixed linear regression analyses, with the results presented in Tables 3 and 4. While several variables showed statistically significant associations in the univariate models, only a subset remained significant in multivariate analyses. The following sections present the variables that retained their significance in the final models.

**TABLE 3:** Univariate and multivariate mixed linear regressions for determinants of perceived appropriateness of coercive measures.

Variable	Category (description)	Univariate linear regression			Multivariate linear regression		
		*β*	95% CI	*p*	*β*	95% CI	*p*
Age	Continuous variable	−0.014	−0.021 to −0.007	**< 0.001**	−0.001	−0.014 to 0.011	0.836
Gender	Female vs. Male	0.083	−0.062 to −0.229	0.264			
Marital status	Married vs. Divorced	0.040	−0.149 to 0.231	0.674			
	Married vs. Widow	−0.269	−0.674 to 0.135	0.191			
	Married vs. Single	0.127	−0.037 to 0.291	0.130			
Ethnicity	Caucasian vs. Other	−0.242	−0.674 to 0.189	0.271			
Spiritual belief	Believer vs. Non‐believer	−0.290	−0.458 to −0.123	**0.001**	−0.225	−0.394 to −0.057	**0.009**
Professional role	Nurse vs. Head Nurse	−0.573	−0.755 to −0.391	**< 0.001**	−0.428	−0.664 to −0.192	**< 0.001**
	Nurse vs. Nursing Aide	0.109	0.072 to 0.290	0.237	−0.002	−0.312 to 0.306	0.985
Educational level	Diploma vs. Bachelor	−0.131	−0.312 to 0.049	0.154	−0.152	−0.438 to 0.133	0.295
	Diploma vs. Post‐bachelor	−0.398	−0.591 to −0.204	**< 0.001**	−0.246	−0.563 to 0.070	0.127
Academic education in mental health nursing	No vs. Yes	−0.168	−0.325 to −0.011	**0.036**	−0.051	−0.212 to 0.109	0.529
Non‐academic education in mental health nursing	No vs. Yes	−0.191	−0.417 to 0.034	**0.097**	0.035	−0.198 to 0.268	0.767
Working experience	Continuous variable	−0.015	−0.022 to −0.009	**< 0.001**	−0.006	−0.018 to 0.005	0.304
Working experience in mental health	Continuous variable	−0.012	−0.018 to −0.007	**< 0.001**	−0.004	−0.011 to 0.003	0.279
Working region	North vs. Centre	0.143	−0.040 to 0.327	0.126	0.199	0.013 to 0.385	**0.036**
	North vs. South	0.264	0.100 to 0.428	**0.002**	0.149	−0.023 to 0.322	0.091
Working setting	Acute vs. Other	−0.235	−0.372 to −0.098	**0.001**	−0.248	−0.415 to −0.080	**0.004**
Contract type	Permanent vs. Fixed‐term	0.406	−0.013 to 0.826	**0.058**	−0.002	−0.427 to 0.423	0.992
Work schedule	Full‐time vs. Part‐time	0.111	−0.268 to 0.499	0.555			
Work pattern	Shift‐work vs. Daytime‐work	−0.254	−0.389 to −0.118	**< 0.001**	0.190	0.007 to 0.373	**0.042**
Perceived work safety	Continuous variable	−0.102	−0.164 to −0.041	**0.001**	−0.015	−0.087 to 0.056	0.680
Perceived job satisfaction	Continuous variable	−0.068	−0.134 to −0.003	**0.040**	−0.019	−0.105 to 0.066	0.659
Intention to change the current work unit	Continuous variable	0.060	0.010 to 0.111	**0.019**	0.020	−0.037 to 0.078	0.479
Intention to leave the profession	Continuous variable	0.006	−0.042 to 0.055	0.788			
Depression	Continuous variable	0.008	−0.000 to 0.016	**0.052**	−0.008	−0.023 to 0.006	0.256
Anxiety	Continuous variable	0.013	0.002 to 0.023	**0.014**	−0.000	−0.017 to 0.015	0.908
Stress	Continuous variable	0.010	0.002 to 0.018	**0.008**	0.012	−0.000 to 0.025	0.060
Stigma toward mental illness	Continuous variable	0.020	0.010 to 0.030	**< 0.001**	0.014	0.003 to 0.025	**0.012**
Humanization of care	Continuous variable	−0.003	−0.011 to 0.005	0.482	0.000	−0.009 to 0.011	0.859

*Note:* Variables with *p* ≤ 0.10 in the univariate model were included in the multivariate model. Bold values indicate statistically significant values.

**TABLE 4:** Univariate and multivariate mixed linear regressions for determinants of perceived appropriateness of security technologies.

Variable	Category (description)	Univariate linear regression			Multivariate linear regression		
		*β*	95% CI	*p*	*β*	95% CI	*p*
Age	Continuous variable	−0.011	−0.018 to −0.003	**0.003**	−0.002	−0.016 to 0.010	0.701
Gender	Female vs. Male	−0.205	−0.356 to −0.055	**0.007**	−0.217	−0.365 to −0.069	**0.004**
Marital status	Married vs. Divorced	−0.094	−0.291 to 0.103	0.349			
	Married vs. Widow	−0.152	−0.570 to 0.265	0.474			
	Married vs. Single	−0.019	−0.189 to 0.149	0.817			
Ethnicity	Caucasian vs. Other	−0.105	−0.550 to 0.340	0.644			
Spiritual belief	Believer vs. Non‐believer	−0.262	−0.436 to −0.089	**0.003**	−0.104	−0.277 to 0.068	0.237
Professional role	Nurse vs. Head Nurse	−0.445	−0.636 to −0.255	**< 0.001**	−0.244	−0.477 to −0.011	**0.040**
	Nurse vs. Nursing Aide	0.072	−0.117 to 0.261	0.454	−0.000	−0.315 to 0.315	1.000
Educational level	Diploma vs. Bachelor	−0.009	−0.197 to 0.177	0.917	0.043	−0.249 to 0.336	0.772
	Diploma vs. Post‐bachelor	−0.290	−0.490 to −0.089	**0.005**	−0.125	−0.446 to 0.195	0.442
Academic education in mental health nursing	No vs. Yes	−0.113	−0.275 to 0.048	0.169			
Non‐academic education in mental health nursing	No vs. Yes	−0.237	−0.470 to −0.004	**0.046**	0.026	−0.213 to 0.267	0.826
Working experience	Continuous variable	−0.011	−0.017 to −0.005	**< 0.001**	0.003	−0.008 to 0.015	0.581
Working experience in mental health	Continuous variable	−0.017	−0.023 to −0.011	**< 0.001**	−0.017	−0.025 to −0.009	**< 0.001**
Working region	North vs. Centre	0.084	−0.103 to 0.273	0.378	0.065	−0.121 to 0.253	0.491
	North vs. South	0.354	0.186 to 0.522	**< 0.001**	0.337	0.159 to 0.516	**< 0.001**
Working setting	Acute vs. Other	0.154	0.011 to 0.296	**0.034**	0.271	0.122 to 0.419	**< 0.001**
Contract type	Permanent vs. Fixed‐term	0.325	−0.108 to 0.758	0.141			
Work schedule	Full‐time vs. Part‐time	0.225	−0.169 to 0.621	0.263			
Work pattern	Shift‐work vs. Daytime‐work	−0.017	−0.158 to 0.123	0.804			
Perceived work safety	Continuous variable	−0.079	−0.143 to −0.016	**0.014**	−0.042	−0.112 to 0.027	0.234
Perceived job satisfaction	Continuous variable	−0.031	−0.098 to 0.036	0.366			
Intention to change the current work unit	Continuous variable	0.045	−0.006 to 0.097	**0.089**	0.012	−0.042 to 0.068	0.648
Intention to leave the profession	Continuous variable	−0.024	−0.074 to 0.026	0.346			
Depression	Continuous variable	0.005	−0.003 to 0.013	0.239	−0.010	−0.025 to 0.004	0.155
Anxiety	Continuous variable	0.015	0.004 to 0.026	**0.005**	0.010	−0.006 to 0.027	0.222
Stress	Continuous variable	0.008	0.000 to 0.016	**0.035**	0.012	−0.000 to 0.025	0.060
Stigma toward mental illness	Continuous variable	0.007	−0.003 to 0.018	0.172	0.001	−0.010 to 0.013	0.787
Humanization of care	Continuous variable	0.001	−0.006 to 0.010	0.677	0.005	−0.005 to 0.015	0.326

*Note:* Variables with *p* ≤ 0.10 in the univariate model were included in the multivariate model. Bold values indicate statistically significant values.

#### 3.2.1 Determinants of Perceived Appropriateness of Coercive Measures

Participants who reported no spiritual belief rated CMs as significantly less appropriate than those who identified with a spiritual belief (*β* = −0.225, 95% CI [−0.394, −0.057], *p* = 0.009). Professional role was also a significant predictor: head nurses rated CMs as less appropriate than registered nurses (*β* = −0.428, 95% CI [−0.664, −0.192], *p* ≤ 0.001). The geographic region also played a role, with participants working in Central Italy perceiving CMs as more appropriate than those in Northern Italy (*β* = 0.199, 95% CI [0.013, 0.385], *p* = 0.036). Work setting significantly influenced perceptions: MHNs employed in acute psychiatric units perceived CMs as more appropriate than those working in non‐acute settings (*β* = −0.248, 95% CI [−0.415, −0.080], *p* = 0.004). Similarly, work patterns had an effect, with daytime workers rating CMs as more appropriate than those working rotating shifts (*β* = 0.190, 95% CI [0.007, 0.373], *p* = 0.042). Finally, stigma scores were positively associated with the perceived appropriateness of CMs, indicating that higher levels of stigma corresponded to a greater endorsement of these measures (*β* = 0.014, 95% CI [0.003, 0.025], *p* = 0.012).

#### 3.2.2 Determinants of Perceived Appropriateness of Security Technologies

Gender was a significant determinant of the perceived appropriateness of STs, with female participants rating them as more appropriate than male participants (*β* = −0.217, 95% CI [−0.365, −0.069], *p* = 0.004). Professional role also influenced perceptions, as head nurses reported lower appropriateness ratings for STs than registered nurses (*β* = −0.244, 95% CI [−0.477, −0.011]; *p* = 0.040). An inverse association was observed between mental health work experience and perceived appropriateness of STs: MHNs with more years of experience in the field evaluated STs as less appropriate (*β* = −0.017, 95% CI [−0.025, −0.009], *p* ≤ 0.001). The geographic region was another relevant factor. Participants working in Southern Italy perceived STs as more appropriate than those in Northern regions (*β* = 0.337, 95% CI [0.159, 0.516], *p* ≤ 0.001). Finally, the setting influenced perceptions: MHNs working in non‐acute environments assigned higher appropriateness ratings to STs than those in acute care settings (*β* = 0.271, 95% CI [0.112, 0.419], *p* ≤ 0.001).

## 4 Discussion

This study is, to the best of our knowledge, the first national investigation of Italian MHNs' perceptions of the appropriateness of CMs and STs in psychiatric settings. By examining both traditional and technological strategies and exploring the influence of personal, professional, and psychosocial factors, this study provides a comprehensive overview of how these measures are evaluated in clinical practice.

Overall, CMs were perceived as moderately appropriate, with significant differences between specific interventions. Locked‐door policies and pharmacological restraints were rated as more appropriate than physical restraints. This pattern is consistent with previous research highlighting a growing critical stance among MHNs toward physical restraint (Doedens et al. 2020; Laukkanen et al. 2020; Wong and Bressington 2022), which is recognized as a last‐resort option (Doedens et al. 2020) due to its ethical, legal, and clinical implications (Chieze et al. 2021; Cusack et al. 2018; Kersting et al. 2019). In contrast, the higher acceptance of pharmacological restraint may reflect its widespread use in psychiatric practice (Belayneh et al. 2024) and greater acceptability among staff (Doedens et al. 2020). Similarly, locked‐door policies were rated as moderately appropriate, likely because of their perceived effectiveness in enhancing ward safety, preventing absconding, and avoiding more invasive interventions (Bowers et al. 2010). In Italy, where closed wards remain prevalent (De Girolamo et al. 2007), such practices may be institutionalized, reinforcing their perceived appropriateness. Moreover, prior research suggests that familiarity and exposure to specific measures foster favorable attitudes (Bowers et al. 2007), and MHNs working in locked environments are more likely to endorse restrictive practices (Bowers et al. 2010).

On average, STs were perceived as slightly more appropriate than CMs, with fixed and portable alarms receiving the highest ratings, followed by CCTV. These technologies may be considered useful for ensuring immediate protection during incidents, consistent with research identifying personal safety as a key concern for psychiatric staff (Delaney and Johnson 2014; Doedens et al. 2020). Their longstanding presence in several psychiatric settings (Anastasi and Bambi 2023; Marhoon et al. 2019), along with national policies promoting their use for violence prevention in healthcare (Italian Ministry of Health 2007), may further contribute to their perceived appropriateness. Conversely, BWCs and metal detectors were rated as less appropriate, reflecting greater hesitation regarding patient‐monitoring tools. Although intended to enhance safety, these tools may raise concerns about privacy violations, potential misuse, and erosion of the therapeutic relationship (Anastasi and Bambi 2023; Foye et al. 2024; Revel et al. 2024), which may explain the lower ratings. These findings suggest that MHNs differentiate between technologies: those facilitating immediate staff protection are viewed more positively than those designed for incident prevention or patient monitoring.

Gender significantly influenced the perceived appropriateness of STs, with males rating them as less appropriate than females. This difference may be interpreted through the lens of social role theory (Eagly and Wood 2016), which posits that social norms shape the roles and behaviors of individuals. In psychiatric settings, these expectations translate into gendered divisions of labour (Torkelson and Seed 2011), with male staff often expected to manage physical confrontations or aggressive behavior to protect female colleagues (Evans and Frank 2003). Empirical studies support this dynamic, showing that male MHNs are more likely to be directly involved in the application of coercion (Wong and Bressington 2022) and adopt a task‐oriented approach to care (Evans and Frank 2003). Consequently, STs might be perceived by male MHNs as undermining their traditional protective role or as a substitute for direct action, potentially explaining their lower perceived appropriateness.

Spiritual beliefs also shaped perceptions, with non‐believers rating CMs as significantly less appropriate than those with spiritual affiliations. This finding supports the notion that spirituality affects ethical reasoning and clinical decision‐making, particularly in morally complex situations, such as the use of coercion (Hem et al. 2018). Spiritual worldviews influence individuals' values and can inform healthcare professionals' clinical judgments and practices (Palmer Kelly et al. 2020). However, the relationship between spirituality and coercion is complex. While spiritual beliefs may promote compassionate interventions, they can also justify coercion through paternalistic reasoning, framing it as a protective act (Hem et al. 2018). Historically, religious justifications have been used to legitimize coercion in psychiatry (Szasz 2007), and such frameworks may still inform contemporary clinical reasoning in Italy.

Professional role was a significant predictor, with head nurses consistently rating both CMs and STs as less appropriate than registered nurses. This aligns with the literature indicating that nurse managers adopt more critical perspectives toward coercion (Laukkanen et al. 2020). A plausible explanation for this may lie in their broader responsibilities. Head nurses are typically tasked with managing clinical risks (Varpula et al. 2024) and ensuring that clinical practice complies with evidence‐based guidelines (Sjølie et al. 2020). These responsibilities may increase their awareness of the legal, professional, and moral implications associated with coercive or technological measures, explaining their lower perceived appropriateness.

Work experience in mental health predicted a lower perceived appropriateness of STs. Previous research has reported a general hesitancy among nurses to adopt technological innovations (Shulzhenko and Holmgren 2020). This pattern aligns with the status quo bias, a cognitive tendency to favor existing routines and resist change (Parker 2021). Considering the recent introduction of many STs into psychiatric care (Anastasi and Bambi 2023), experienced MHNs may approach the use of technology with skepticism, potentially perceiving it as less appropriate within their established framework of care.

The geographic region was also a significant determinant. Compared to MHNs in the North, those in Central Italy rated CMs as more appropriate, while those in the South perceived STs favorably. These differences likely reflect broader systemic disparities in healthcare infrastructure, resource allocation, and uneven implementation of psychiatric reforms across the country (De Girolamo et al. 2007). Informed by the national psychiatric reform (Basaglia Law), Northern Italy promotes restraint‐free community‐based care models and hosts most no‐restraint psychiatric units (Pocobello et al. 2024), potentially contributing to more critical views of CMs. Moreover, the national psychiatric reform has often lacked uniform financial and organizational support, leading to its heterogeneous implementation across the country (Mezzina 2018). In under‐resourced areas, such as parts of Southern Italy, STs may be perceived as an appropriate solution to address workforce shortages, safety risks, or infrastructure constraints. Furthermore, local organizational cultures and traditions may shape perceptions of safety measures (Bowers et al. 2007).

The work setting significantly influenced the perceived appropriateness. MHNs in non‐acute settings rated CMs as less appropriate and STs as more appropriate than those in acute settings. These differences likely reflect the distinct clinical demands, patient profiles, and safety challenges of each of these settings. Acute psychiatric units care for individuals in severe mental health crises (Bowers et al. 2009) and face higher rates of violence (d'Ettorre and Pellicani 2017; Odes et al. 2021). Exposure to violence is a known factor leading to CMs' use (Doedens et al. 2020; Happell and Harrow 2010). In these high‐risk environments, MHNs may view CMs as necessary for managing aggression (Happell and Harrow 2010) and maintaining safety (Doedens et al. 2020; Wong and Bressington 2022), leading to more favorable attitudes toward their use. In contrast, MHNs in non‐acute settings are less exposed to immediate threats and focus more on long‐term therapeutic relationships with patients. In these contexts, STs may be perceived as practical tools to enhance safety and compensate for limited staff.

Work patterns also shaped MHNs' perceptions of CMs, with those on daytime shifts rating them as more appropriate than those on rotating shifts. This aligns with research showing that the use of CMs varies across shifts (Doedens et al. 2020) and that rotating shifts can influence staff attitudes toward the use of coercion (Wong and Bressington 2022). Some forms of coercion are used less frequently at night (Wong and Bressington 2022), and they are often initiated during the day in anticipation of reduced night staffing (Yang et al. 2014). These patterns suggest that daytime MHNs, who are more often involved in initiating CMs, may perceive them as more appropriate because of their direct engagement in their application.

Finally, higher stigma levels were associated with greater perceived appropriateness of CMs. This supports previous research indicating that stigmatizing attitudes in the general population predict stronger support for coercion in psychiatric care (Steiger et al. 2023). Stigma remains a relevant factor that influences professionals' views on coercion (Gaebel and Zäske 2011). Stigma operates through a process of labelling, stereotyping, and social distancing that reinforces power imbalances (Andersen et al. 2022). In psychiatric settings, where MHNs often hold positions of authority and control (Bowers et al. 2009), these dynamics may be amplified, contributing to the greater perceived appropriateness of CMs.

### 4.1 Limitations and Future Research

Despite the large national sample, which enhances the generalisability of the findings, some methodological limitations should be acknowledged. First, the cross‐sectional design precludes causal inferences, and self‐reported measures may have introduced response bias. Second, the assessment focused on perceived appropriateness, which may not fully reflect actual clinical behaviors. Third, the assessment captured general perceptions of appropriateness and did not differentiate between specific clinical scenarios, which may influence evaluations. Fourth, there were missing responses, primarily observed in sensitive measures, such as psychological distress and stigma. This pattern is common in surveys involving potentially stigmatizing or sensitive content. Consequently, individuals experiencing greater psychological burden, higher stigma, or more polarized views toward CMs and/or STs may have been slightly underrepresented. Finally, although the overall geographic distribution is closely aligned with national workforce estimates, minor subgroup deviations were observed, particularly among nursing aides, which should be considered when interpreting role‐specific findings. Nevertheless, the proportion of excluded cases was small (7.5%), and the final sample substantially exceeded the minimum required size, reducing the likelihood that these limitations significantly affected the study's main findings.

Future research should explore longitudinal changes in MHNs' perceived appropriateness of CMs and STs, particularly in response to educational interventions, policy changes, or organizational innovations. Moreover, considering that perceived appropriateness may vary depending on the reason for use (e.g., risk of violence vs. self‐harm), future studies should incorporate scenario‐based assessments to capture this nuance. Qualitative studies may also provide deeper insights into the subjective meanings, ethical tensions, and related dynamics that influence MHNs' perceptions and decision‐making in clinical practice. Comparative research across countries could further elucidate how structural, cultural, and policy‐related factors shape perceptions and practices related to safety in psychiatric care. Finally, with the increasing adoption of technologies in mental health, further investigation is warranted into the ethical, practical, and therapeutic implications of these devices, particularly in terms of how they may affect the experiences of staff and service users.

## 5 Conclusion

This national study provides a comprehensive overview of Italian MHNs' perceptions of the appropriateness of CMs and STs in psychiatric settings. Although no intervention was deemed inappropriate on average, relevant differences were observed. Among CMs, pharmacological restraint and locked‐door policies were rated as more appropriate than physical restraints. Similarly, alarms and CCTV were the most endorsed compared to BWCs and metal detectors.

A key finding was the variation in perceptions across professional roles. Head nurses consistently expressed more critical views than registered nurses and nursing aides, suggesting heightened ethical sensitivity and awareness of the broader implications of using these interventions. This pattern highlights the potential of head nurses to act as catalysts for change within mental health services, promoting ethical reflection, evidence‐based practice, and restraint reduction.

MHNs' insights were shaped by a complex interplay of sociodemographic, professional, and contextual factors. Perceptions of CMs' appropriateness were influenced by their spiritual beliefs, geographic location, work setting, shift pattern, professional role, and levels of stigma. Views on STs were influenced by gender, professional role, years of mental health experience, geographic location, and the work setting. These findings illustrate that evaluations of safety practices are not merely clinical judgments but are embedded in broader personal, cultural, and institutional contexts.

Such complexity highlights the need for structured, specialized training for MHNs that extends beyond technical competence and includes the ethical, psychological, and relational dimensions of care. Educational programs should cultivate critical awareness and provide staff with the tools to evaluate the use of coercive and technological interventions within a human rights framework. Training should also be adapted to different professional roles and levels of experience to ensure relevance across diverse psychiatric settings and MHNs.

At the policy level, the use of CMs and STs should not be driven by gaps in staffing, resources, or infrastructure. These measures must not serve as substitutes for systemic investment. Adequate funding for mental health services is essential to ensure that safety practices are implemented judiciously and only when appropriate, within therapeutic environments that support recovery and dignity.

Moreover, the inclusion of anti‐stigma education and bioethics in both pre‐registration and continuing professional development is fundamental to achieving this goal. Addressing stigma within the profession can reduce support for coercive practices and foster a culture of empathy, respect, and patient‐centred care. Bioethics education can strengthen clinical reasoning and guide decision‐making in ethically complex situations.

Finally, the voices of MHNs must be actively included in shaping organizational policies and national mental health care strategies. As frontline professionals, their insights are relevant for understanding the real‐world implications of safety interventions and ensuring that reforms are grounded in both evidence and experience. Their involvement is essential for the legitimacy of policy decisions and their successful implementation in the field.

Future research should explore how these perceptions evolve over time and in response to targeted interventions or organizational changes. Longitudinal and qualitative studies, especially those involving patients, families, and interdisciplinary teams, are crucial for advancing safer, ethical, and more appropriate models of psychiatric care.

Ultimately, enhancing safety in mental health settings requires more than just the use of procedures or technologies. It needs a sustained commitment to balance protection with dignity, an equilibrium that can only be achieved through critical reflection, inclusive dialogue, and shared responsibility among all stakeholders involved in mental health care.

## Funding

The authors have nothing to report.

## Disclosure

Institutional Review Board Statement: The study was approved by the Local Ethics Committee of Palermo 1 (protocol no. 204/2024, dated 28 March 2024).

Clinical Resources Section: World Psychiatric Association (WPA). Supporting and implementing alternatives to coercion in mental health care (https://www.wpanet.org/alternatives‐to‐coercion). World Health Organization (WHO). WHO QualityRights Specialized training: Strategies to end seclusion and restraint (https://www.who.int/publications/i/item/who‐qualityrights‐guidance‐and‐training‐tools).

## Ethics Statement

This study adhered to international and national ethical standards, with formal approval granted by the Local Ethics Committee of Palermo 1 (protocol no. 204/2024, dated 28 March 2024). The study was conducted following the principles of the Declaration of Helsinki, Good Clinical Practice (Ministerial Decree 15/07/1997 and subsequent amendments), and the Oviedo Convention. Additionally, this study complied with Italian regulations governing non‐interventional research, including the General Data Protection Regulation (GDPR) 2016/679 and the Italian Personal Data Protection Code (Legislative Decree 101/2018). Participants received detailed information about the study objectives, procedures, potential risks and benefits, and their rights. Key assurances included anonymity, confidentiality, voluntary participation, and the ability to withdraw at any time without consequences. This information was clearly communicated in the invitation letter and repeated on the survey's homepage. Informed consent was obtained before participation, following the Italian Legislative Decree 196/2003 and the Declaration of Helsinki. All questionnaires were completed anonymously, and no personally identifiable information was collected. Data was securely stored on a password‐protected server, and access was restricted to authorized research team members according to the current body of laws on privacy. All analyses were conducted on aggregated data, ensuring that no individual participant or institution could be identified. The study posed no physical or psychological risks to the participants and did not involve minors. No financial incentives or rewards were provided to the participants or researchers. All ethical and privacy protection guidelines were meticulously followed to ensure the integrity of the research.

## Consent

Informed consent was obtained from all participants involved in the study.

## Conflicts of Interest

The authors declare no conflicts of interest.

## Supporting information


**Data S1:** STROBE Statement—Checklist of items that should be included in reports of *cross‐sectional studies*.

## Data Availability

The data that supports the findings of this study are available on request from the corresponding author. The data are not publicly available due to privacy or ethical restrictions.
